# The Long-Term Effect of Smoking on 10 Years’ Survival and Success of Dental Implants: A Prospective Analysis of 453 Implants in a Non-University Setting

**DOI:** 10.3390/jcm9041056

**Published:** 2020-04-08

**Authors:** Simon Windael, Stijn Vervaeke, Stefanie De Buyser, Hugo De Bruyn, Bruno Collaert

**Affiliations:** 1 School of Dental Medicine, Department of Periodontology and Oral Implantology, Faculty of Medicine and Health Sciences, Ghent University, 9000 Ghent, Belgium; stijn.vervaeke@ugent.be (S.V.); hugo.debruyn@ugent.be (H.D.B.); 2 Private Practice Periodontology and Oral Implantology, 8940 Geluwe, Belgium; 3 Biostatistics Unit, Faculty of Medicine and Health Sciences, Ghent University, 9000 Ghent, Belgium; Stefanie.DeBuyser@ugent.be; 4 Radboud Institute for Health Sciences, Department of Dentistry—Implantology & Periodontology, Radboud University Medical Center, 6525 GA Nijmegen, The Netherlands; 5 Center for Periodontology and Implantology Leuven, 3001 Heverlee, Belgium; collaert@paro-implanto.be

**Keywords:** smoking, dental implants, crestal bone loss, marginal bone loss, long-term survival

## Abstract

Background: The purpose of this study was to compare the survival and peri-implant bone loss of implants with a fluoride-modified surface in smokers and non-smokers. Material and Methods: All patients referred for implant treatment between November 2004 and 2007 were scrutinized. All implants were placed by the same surgeon (B.C.). The single inclusion criterion was a follow-up time of at least 10 years. Implant survival, health, and bone loss were evaluated by an external calibrated examiner (S.W.) during recall visits. Radiographs taken at recall visits were compared with the post-surgical ones. Implant success was based on two arbitrarily chosen success criteria for bone loss (≤1 mm and ≤2 mm bone loss after 10 years). Implant survival in smokers and non-smokers was compared using the log-rank test. Both non-parametric tests and fixed model analysis were used to assess bone loss in both groups. Results: A total of 453 implants in 121 patients were included for survival analysis, and 397 implants in 121 patients were included for peri-implant bone-loss analysis. After a mean follow-up time of 11.38 years (SD 0.78; range 10.00–13.65), 33 implants out of 453 initially placed had failed in 21 patients, giving an overall survival rate of 92.7% and 82.6% on the implant and patient level, respectively. Cumulative 10 years’ survival rate was 81% on the patient level and 91% on the implant level. The hazard of implant loss in the maxilla was 5.64 times higher in smokers compared to non-smokers (*p* = 0.003). The hazard of implant loss for implants of non-smokers was 2.92 times higher in the mandible compared to the maxilla (*p* = 0.01). The overall mean bone loss was 0.97 mm (SD 1.79, range 0–17) at the implant level and 0.90 mm (SD 1.39, range 0–7.85) at the patient level. Smokers lost significantly more bone compared to non-smokers in the maxilla (*p* = 0.024) but not in the mandible. Only the maxilla showed a significant difference in the probability of implant success between smokers and non-smokers (≤1 mm criterion *p* = 0.003, ≤2 mm criterion *p* = 0.007). Taking jaw into account, implants in smokers experienced a 2.6 higher risk of developing peri-implantitis compared to non-smokers (*p* = 0.053). Conclusion: Dental implants with a fluoride-modified surface provided a high 10 years’ survival with limited bone loss. Smokers were, however, more prone to peri-implant bone loss and experienced a higher rate of implant failure, especially in the upper jaw. The overall bone loss over time was significantly higher in smoking patients, which might be suggestive for a higher peri-implantitis risk. Hence, smoking cessation should be advised and maintained after implant placement from the perspective of peri-implant disease prevention.

## 1. Introduction

One in five adults in the world smokes tobacco, despite the fact that negative effects on oral and general health are well known. Every seventh death in the world (13%) was the result of direct smoking in 2017; a further 2% was the result of secondhand smoke. This means 15%—close to 1-in-6 deaths—was the result of tobacco. However, since 1990, there is a declining global trend in smoking reflected almost everywhere across the world [1,2,3,4]. Smoking is associated with various serious health conditions, such as cancer, respiratory problems, and cardiovascular diseases, but can also lead to sleep deficiency and depression [5,6]. Smoking shows an overwhelmingly negative influence on oral health, affecting both soft and hard tissues. It is known as an important risk indicator for poor oral wound healing, dry socket, implant failure, and marginal bone loss around teeth and implants [5,7,8,9,10,11,12,13,14,15,16,17,18,19,20,21,22,23]. In regard to dental implants, a significant relationship has been shown between smoking and the risk of failure of osseointegrated implants, more particularly in the upper jaw [8,24]. Smoking seems to have an early effect on osseointegration, dependent on the properties of the implant surface and local host genetic responses. It is also suggested that smokers, compared to non-smokers, have an altered bone structure and composition [25,26]. Using multilevel analysis, including early as well as late implant loss, smoking has been associated with a significantly higher percentage of early lost implants (2.2%) in comparison to non-smoking (0.9%). Late implant failure seems not to be affected by smoking habits [27]. A systematic review has shown a higher risk for implant failure in smokers with a patient-related odds ratio of 2.64 and an implant-related odds ratio of 2.25 [28]. Another systematic review shows an average implant survival ranging between 65.3%–97% for smokers versus 82.7%–98.8% for non-smokers. A statistically significant difference in favor of non-smokers has also been found with an OR of 1.96 for implant failure [29]. Cigarette smoking is associated with a reduction in bone mineral density in a dose-related and duration-related manner [7,30]. A higher incidence of marginal bone loss is found for smokers with subsequent years [31]. Smokers show more than two times greater marginal bone loss and more than three times greater risk for implant loss in the maxilla [32]. Vervaeke et al. (2015) showed an estimated additional bone loss of 1.18 mm for smokers vs. non-smokers [18]. A uni- and multivariate analysis has identified smoking as a significant factor affecting implant treatment outcomes, especially in the maxilla.

Over the last decade, implants surfaces have been modified from smooth/rough to moderately rough surface texture, expressed by an average Sa value of 1–2 µm [33,34,35]. This evolution in surface topography has positively affected the bone-to-implant contact, even in smoking patients [36]. In 2004, a fluoride-modified surface was introduced (OsseospeedTM, Dentsply, Astra Tech, Mölndal, Sweden), with a moderately rough surface with nanoscale topography (Sa value of (1.32–1.82 µm)). A number of animal and human studies have been carried out to evaluate clinical performance. The results have suggested that osseointegration has been enhanced (especially during the first weeks of healing), by enhanced osteoblast differentiation, platelet activation, and surface thrombogenic and osteoconductive properties [37,38,39,40]. This attributes to improved survival rate, esthetic outcome, and marginal bone remodeling [41]. Even more challenging situations show good short term results with limited marginal bone loss and high implant survival like heavy alveolar atrophied ridges with augmentation [41,42,43,44] and smoking patients [36].

The purpose of the present study was to evaluate the 10 years’ survival and success of implants with a fluoride-modified surface in smokers and non-smokers treated under daily clinical and non-specifically selected conditions.

## 2. Materials and Methods

### 2.1. Patient Selection and Clinical Procedure

All patients in need of implant placement between November 2004 and 2007 were evaluated. During intake and at the 10-year follow-up session, a medical history was taken, including self-reporting of smoking habits. The initial 2-years report was presented previously [32]. The same surgeon (BC) placed all implants in healed ridges. No bone grafting, sinus lift, or guided bone regeneration procedures were used. Implants were placed using different surgical techniques (one-stage and two-stage surgery) and different loading protocols (immediate versus delayed loading). Hence, 3 types of protocols were performed: immediate loading, one-stage delayed loading, and two-stage delayed loading. Surgery consisted of a crestal incision, followed by full mucoperiosteal flap elevation, implant installation (Osseospeed^TM^, Dentsply, Astra Tech, Mölndal, Sweden), following the manufacturer’s guidelines and suturing. Implant installation was immediately followed by radiographs (baseline) with commercially available film-holders (Uni-Bite Film Holder^TM^, Dentsply, York, PA, USA) using the parallel long-cone technique to visualize marginal bone-to-implant contact points and implant threads. Care was taken to shoot perpendicular on the implant axis. The individualization of standard film holders was not manageable in private practice. If implants threads were not clear, the radiograph was discarded, and a new radiograph was taken. To determine marginal bone levels correctly, the digital images were magnified by the software. Possible distortions were solved by calibration, based on the known abutment height and implant diameter. There might be a slight difference in the horizontal plane, but the evaluated implants were round, so this difference should be rather limited.

Hence, bone loss beyond the reference point was reported from the time of surgery, and initial bone remodeling was included in the total bone level changes over time. After the final restorations were made by the referring dentist, a professional maintenance schedule (including radiographic follow-up) was proposed to each patient, whereby the frequency was based on the clinical situation and individual needs. Given the fact that the patients were referred by and, therefore, returned to their original dentist, only patients that maintained their visits at the specialist clinic were included in the current study. These patients were prospectively followed up for at least 10 years. Briefly, this consisted of a recall interval of 6 or 12 months during the first 2 years and 12 or 24 months during the following years. All implants with at least 10 years of follow-up and part of the professional maintenance recall system of the specialist center were included to evaluate implant survival and peri-implant bone loss. An independent external examiner (SW) from the University of Ghent performed the recall consultation at the 10 years’ follow-up and had access to the patient files. All patients were thoroughly informed and signed a written consent form. The study protocol was approved by the ethical committee of the Ghent University Hospital (Ghent, Belgium) under number B670201524796.

### 2.2. Examination Criteria

**Figure 1:**
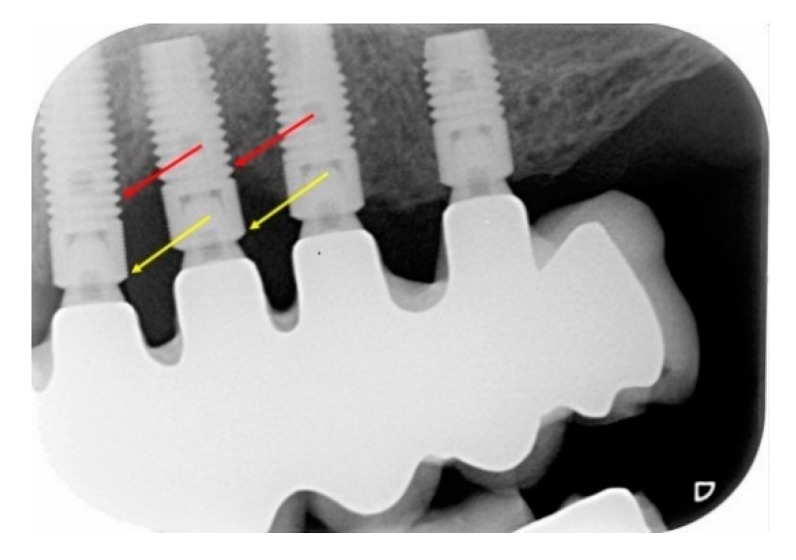
The yellow arrow points to the reference point (lower border of the smooth implant collar). The red arrow shows the first bone-to-implant contact. The distance in between was measured with digital software.

Smoking was defined as the smoking of at least 1 cigarette a day and was based on self-reporting. Ex-smokers and non-smokers were combined into the group of non-smokers. A history of periodontitis was based on the following criteria: (a) radiographic proof of bone loss extending 33% of the root length of residual teeth at the time of referral; (b) patients who were treated with (non)surgical periodontal treatment before implant therapy; (c) when before implant treatment, hopeless teeth were extracted due to periodontitis; (d) edentulous patients with evidence of periodontitis at the time of referral based on radiographs obtained in retrospect from the referring dentist. Peri-apical radiographs were analyzed with the use of digital Software (Visi-Quick®, Amsterdam, The Netherlands) with an accuracy of 0.1 mm. These were taken from the day of surgery up to at least 10 years in function. The crestal bone level was calculated at both mesial and distal sites of each implant by measuring the distance between the reference point (lower border of the smooth implant collar) to the first marginal bone-to-implant contact (Figure 1).

Bone loss beyond the reference point was calculated by comparing peri-apical radiographs taken during recall visits after 3 months, 1, 2, and 10 years with baseline (implant installation). The mean of both bone level readings (mesial and distal) was calculated to obtain a single value per implant. Plaque- and bleeding assessment was performed at six sites [45]. Pocket probing was performed manually with a periodontal probe (CP 15 UNC, Hu-Friedy Mfg. Co. Inc, Chicago, IL, USA) at 6 sites of the implant, immediately followed by the scoring of bleeding on probing. An implant was considered as a failure when it was removed due to the following reasons: implant mobility, loss of integration, ongoing bone loss, infection, persistent pain, or patient discomfort [46]. An individual implant was considered a success when total bone loss beyond the reference point, from the placement of the implant to 10 years of follow-up, was less than 1 or 2 mm [47,48]. Incidence of peri-implantitis of the implants under maintenance after 10 years was calculated based on the Consensus report of the 4th workgroup of the 2017 World Workshop on the classification of periodontal and peri-implant diseases and conditions, by combining a total bone loss ≥3 mm with increasing probing depth ≥6 mm and bleeding/suppuration on location [49]. The survival of the implant, the peri-implant bone loss, and the pocket probing depth were accounted as the dependent variables.

### 2.3. Statistical Analysis

#### 2.3.1. Implant Survival

For survival analysis at the patient level, we only included patients who had at least one implant with ≥ 10 year-follow-up (*n* = 121). For survival analysis at the implant level, we only included implants from patients who had at least one implant with ≥ 10 year-follow-up and with known observation time (*n* = 453). Kaplan–Meier estimates of implant survival at the patient level were compared between smokers and non-smokers with the log-rank test. The estimated survival rates at 1, 2, 5, and 10 years were reported together with the 95% confidence intervals, which were calculated using the “log-log” approach. Hazard estimates of implant loss at the implant level were compared between smokers and non-smokers, overall and per jaw using the Robust Score test for a simple Cox proportional hazards model. Robust standard errors were estimated to take into account the clustering of implants within patients. The estimated survival rates at 1, 2, 5, and 10 years were reported together with the 95% confidence intervals—calculated using the “log-log” approach. These confidence intervals didn’t take into account the clustered design. A multiple Cox proportional hazards model—for smoking status, jaw, and their two-way interaction—was fitted with robust estimation of the standard error to take into account the clustering of implants within patients. Robust Wald 95% confidence intervals and corresponding *p*-values were reported. Life tables (Table 1, Table 2 and Table 3) show the number of implant loss and the total number of implants at risk for implant loss as well as the cumulative survival rate for each year interval. Those were presented as overall, according to smoking status and according to both smoking status and jaw.

#### 2.3.2. Peri-Implant Bone Loss (mm)

Intra- and inter-examiner reliability was evaluated using the intraclass correlation coefficient (ICC) based on a two-way random model with absolute agreement. The observed mean, standard deviation, minimum, and maximum were used to describe bone loss beyond the reference point in several subgroups at different time points. Cumulative frequencies of bone loss in mm were plotted for different time intervals. Lower curves would have a smaller proportion of bone loss <2 mm than higher curves and, hence, more bone loss. Cumulative frequencies of bone loss in mm were plotted for smokers and non-smokers. For the analyses at the implant level, a linear mixed model for bone loss in mm was fitted with a random intercept for the patient to account for multiple implants within a patient and with smoking status, jaw, and their two-way interaction as fixed effects. Estimated marginal means at the original target scale for smoking status and for smoking status * jaw were requested together with the pairwise comparisons. No test was performed to compare mean bone loss at the patient level between smokers and non-smokers because the residuals were not normally distributed, and, unlike with the analysis at the implant level, one could not solely rely on the central limit theorem due to the smaller sample size. A non-parametric test would compare the mean rank between smokers and non-smokers, instead of comparing the actual mean.

#### 2.3.3. Implant Success

Implant success was defined in two ways: Firstly, as ≤1 mm bone loss after 10 years, and, secondly, as ≤2 mm bone loss after 10 years. For analysis at the implant level, a generalized linear mixed model with a binomial distribution and logit link for implant success was fitted with a random intercept for the patient and with smoking status, jaw, and their two-way interaction as fixed effects. Estimated marginal means at the original target scale for smoking status and for smoking status * jaw were requested together with the pairwise comparisons. For the analyses at the patient level, Fisher’s exact test was used to test for a difference in the proportion of implant success between smokers and non-smokers.

#### 2.3.4. Peri-Implant Health (Implant Level)

Mean bleeding on probing (at the time of recall visit) and mean probing pocket depth were calculated. The possibility of a statistically significant difference was examined by non-parametric testing (Mann–Whitney U test). Peri-implant mucositis was identified as the presence of bleeding and/or suppuration on gentle probing with or without increased probing depth compared to previous examinations and absence of bone loss beyond crestal bone level changes resulting from initial bone remodeling. Peri-implantitis was defined as loss of crestal bone with time ≥3 mm, suppuration, and/or bleeding on probing, with or without increasing probing pocket depth ≥6 mm [49]. A generalized linear mixed model with a binomial distribution and logit link for peri-implantitis was fitted with a random intercept for the patient and with smoking status, jaw, and their two-way interaction as fixed effects. Estimated marginal means at the original target scale for smoking status and for smoking status * jaw were requested together with the pairwise comparisons.

#### 2.3.5. Software

Statistical descriptive analysis and non-parametric testing for patient compliance were performed using SPSS v23 (IBM^®^, Armonk, NY, USA).

All other analyses were performed in R version 3.6.1 using the “lme4” package and the “survival” package (R Foundation for Statistical Computing, Vienna, Austria).

## 3. Results

### 3.1. Patient Population

Of the original 300 patients included in the previous report (Vervaeke et al 2012), 81 patients had never been maintained in the specialist clinic and had returned to their own dentist for regular maintenance, 6 maintained patients had passed away, and 72 had ignored maintenance over time at the specialty clinic and returned to their referring dentist or indicated not to participate in the proposed recall program. In total, 141 patients were cooperative and compliant with the maintenance program and were invited for the research assessment, and 121 responded positively (drop-out 14.2%). Forty-eight were male, and 73 were female, with a mean age of 65.2 years (SD 11; range 31–88). An overview of the distribution of implant length and diameter, with notification of implant loss, is shown in Table 4.

Of the total of 121 patients, 43 had single crowns, 51 had fixed partial dentures, 24 had fixed cross-arch bridges, 2 had overdenture on locators, and 1 patient had an overdenture on a bar-structure. On the implant level, 67 implants supported single crowns, 180 supported fixed partial dentures, 200 supported fixed cross-arch bridges, 4 supported overdentures on locators, and 2 implants supported an overdenture on a bar-structure. Only one patient, a non-smoker, had diabetes (regulated with medication), and one patient started oral bisphosphonates during follow-up, several years after implant treatment. Smokers showed significantly higher compliance compared to non-smokers (*p* = 0.001).

### 3.2. Implants Survival

**Figure 2:**
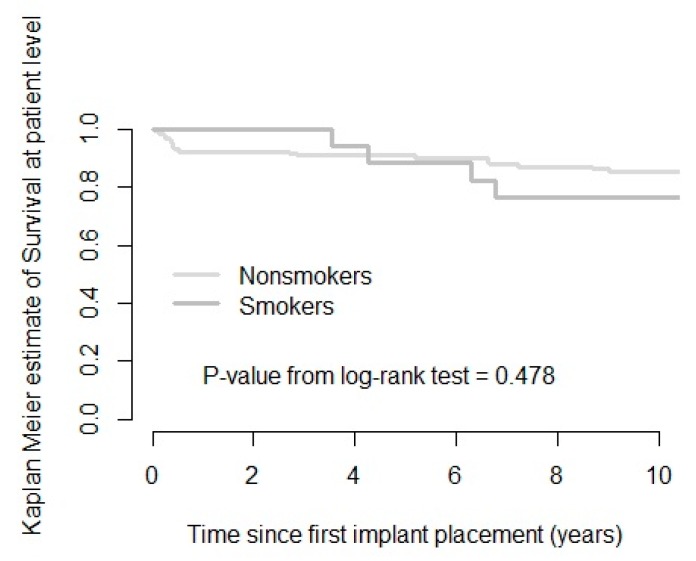
Kaplan–Meier survival curve showing estimated implant failures in the function of time for smokers and non-smokers on the patient level.

**Figure 3:**
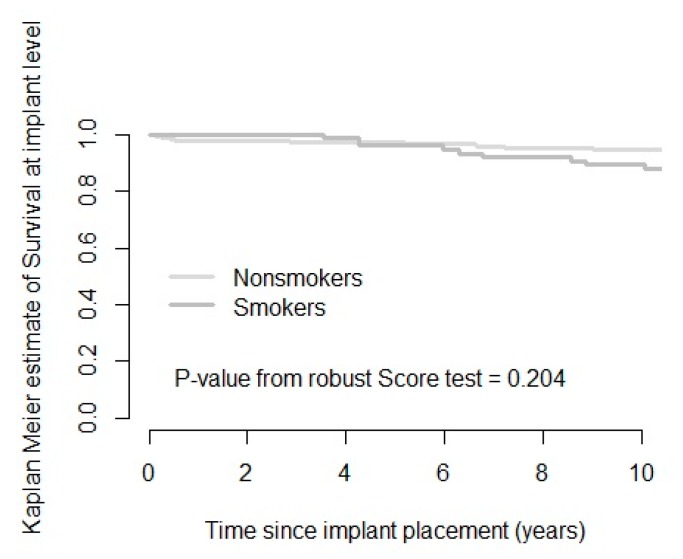
Kaplan–Meier survival curve showing estimated implant failures in the function of time for smokers and non-smokers on the implant level.

After a mean follow-up time of 11.38 years (SD 0.78; range 10.00–13.65), 33 implants out of 453 initially placed had failed in 21 patients. An absolute survival rate of 92.7% and 82.6% on the implant and patient levels was seen, respectively. The cumulative 10 years’ survival rate (CSR) was 81% on the patient level and 91% on the implant level (Figure 2 and Figure 3, Table 1 and Table 2).

Eleven out of 76 implants failed in smokers, and 22/377 in non-smokers, resulting in absolute survival rates of 85.5% and 94.2%, respectively. CSR’s were 82% vs. 75% on the patient level and 93% vs. 81% on the implant level for non-smokers and smokers, respectively. Eight implants failed before prosthetic loading, all in non-smokers.

Regarding the jaw of treatment, 17/272 (6.25%) implants in the upper jaw and 16/181 (8.84%) implants in the lower jaw failed. For smokers, 3/35 (8.57%) implants failed in the mandible and 8/41 (19.51%) in the maxilla. For the non-smoking group, implant failure for the mandible was 13/146 (8.90%) and for the maxilla 9/231(3.9%). CSR’s in respect of smoking status and jaw are mentioned in Table 3. These were 89% vs. 96% for non-smokers and 88% vs. 76% for smokers, respectively, in the mandible and maxilla. No statistical differences were found between smokers and non-smokers regarding survival at the patient level, implant level, or regarding the type of jaw (based on Kaplan–Meier estimate of survival). Only a significant difference was found in non-smokers with a higher survival rate for the maxilla (97% vs. 93% for the mandible, *p* = 0.047). However, the hazard of implant loss for implants of the maxilla was 5.64 times higher in smokers compared to non-smokers (95% CI for the HR went from 1.82 to 17.5) (*p* = 0.003). The hazard of implant loss for implants of non-smokers was 2.92 times higher in the mandible compared to the maxilla (95% CI for the HR went from 1.29–6.62) (*p* = 0.01).

### 3.3. Peri-Implant Bone Loss

**Figure 4:**
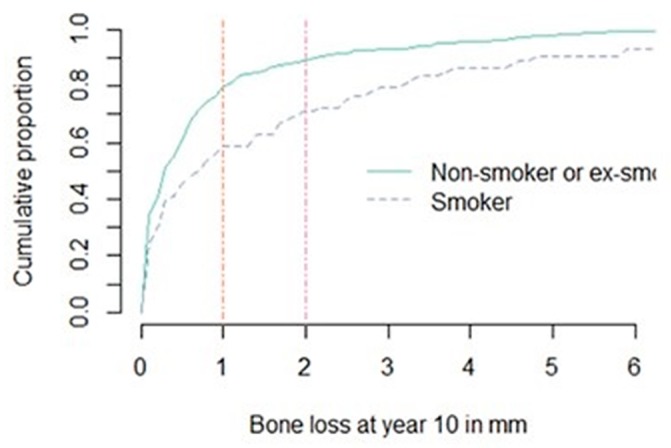
Cumulative percentage of individual peri-implant bone loss, smokers compared to non-smokers.

**Figure 5:**
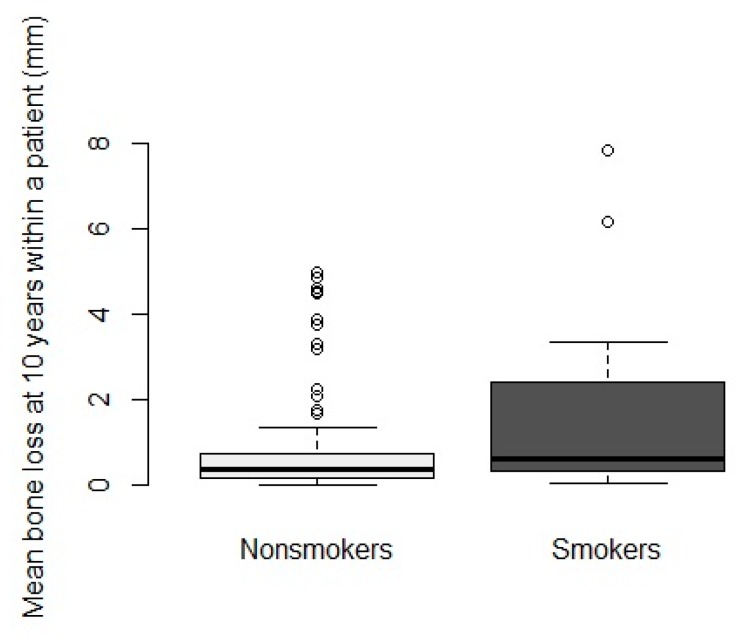
Boxplot reporting on mean peri-implant bone loss within each patient, comparing smokers and non-smokers after at least 10 years.

Regarding the different treatment protocols described, a separate analysis was not considered beneficial. This was to not decrease the power of the study, focusing on smoking habits on the long-term outcome. Another study by Vervaeke and coworkers (2015) found no statistical difference between the three treatment protocols [18]. From the 453 initially placed implants in the followed population, 397 implants in 121 patients had readable radiographs. The intra-examiner repeatability for bone loss was high (ICC 0.99, 95% confidence interval (CI) (0.98–0.99)), as was the inter-examiner repeatability (ICC 0.84, 95% CI (0.76–0.89)). After a mean follow-up time of 11.38 years, mean bone loss beyond the reference point for all cases was 0.97 mm (SD 1.79, range 0–17) at the implant level and 0.90 mm (SD 1.39, range 0–7.85) at the patient level. When comparing smokers and non-smokers irrespective of jaw location, a mean bone loss of 1.93 mm (SE 0.57, 95% CI (0.811–3.047)) and 0.8 mm (SE 0.12, 95% CI (0.556–1.024)) on the implant level and 1.71 mm (SD 2.32, range 0.05–7.85) and 0.77 mm (SD 1.15, range 0–4.97) on the patient level was found, respectively. For mean bone loss according to smoking status adjusted for jaw, there was a significant difference in estimated mean bone loss at 10 year-follow-up between smokers and non-smokers (*p* = 0.0031) with smokers having a higher mean bone loss (1.9 mm versus 0.8 mm, estimated mean difference of 1.12 mm) (Figure 4 and Figure 5).

**Figure 6:**
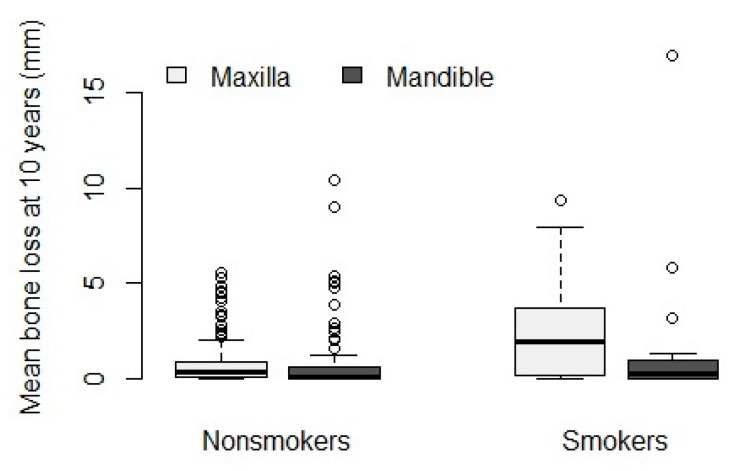
Boxplot reporting on individual peri-implant bone loss in smokers and non-smokers after a minimum of 10 years, comparing upper and lower jaw.

Considering the jaw of treatment in smokers versus non-smokers, mean bone loss of 2.46 mm (SE 0.721, 95 % CI (1.043–3.877)) versus 0.80 mm (SE 0.141, 95% CI (0.522–1.077)) was found for the maxilla. For the mandible, this was 1.28 mm (SE 0.752, 95% CI (−0.194–2.762)) versus 0.78 mm (SE 0.206, 95% CI (0.369–1.180)). Only for the maxilla, the difference of mean bone loss was significant between smokers and non-smokers (*p* = 0.006). The difference in bone loss between maxilla and mandible was not significant within both groups (smoking *p* = 0.47 and non-smoking *p* = 1) (Figure 6).

### 3.4. Implant Success

Implant success was calculated using a threshold for individual total bone loss arbitrary set at ≤1 mm and ≤2 mm changes. This was based on additional bone loss measured between 24 and 120 months. Table 5 gives a summary of the successful implants in smokers and non-smokers with respect to jaw location.

With the success criterion of “bone loss ≤1 mm after 10 years of follow-up”, non-smokers showed 81.2% implant success versus 59.7% for smokers. This difference was significant (*p* = 0.049, adjusted for jaw). There was a significant difference in the probability of implant success between smokers and non-smokers in the upper jaw (78.9% success in non-smokers versus 41.2% success in smokers, *p* = 0.003). In our sample, a significant difference in the probability of implant success between smokers and non-smokers in the lower jaw was absent (84.9% success in non-smokers versus 82.1% success in smokers, *p* = 0.761). When the criterion was defined as “bone loss ≤2 mm after 10 years of follow-up”, non-smokers showed an overall success rate of 88.7% versus 69.4% for the smoking group, not statistically significant (*p* = 0.112, adjusted for jaw). A significant difference in the probability of implant success was seen in the upper jaw (88.5% success in non-smokers versus 52.9% success in smokers, *p* = 0.007). In our sample, one could not find a significant difference in the probability of implant success in the lower jaw (88.9% success in non-smokers versus 89.3% success in smokers, *p* = 0.961). Only the smoking group showed a significant difference in implant success between maxilla and mandible, with higher implant success in the mandible (1 mm criterion *p* = 0.004; 2 mm criterion *p* = 0.015). There was an indication of effect modification of smoking by the jaw, although not statistically significant (*p*-value Fixed effects = 0.081). We found no difference in the proportion of implant success at the patient level between smokers and non-smokers (1 mm criterion *p*-value from Fisher’s exact test = 0.277 and 2 mm criterion *p*-value from Fisher’s exact test = 0.061).

### 3.5. Peri-Implant Health

The overall mean bleeding on probing was 0.30 (SD 0.38, range 0–1), with 30% of the implants showing bleeding on probing, 0.29 (SD 0.38, range 0–1) in non-smokers versus 0.35 (SD 0.41, range 0–1) in smokers (*p* = 0.332). Overall mean pocket probing depth was 4.25 mm (SD 1.26, range 2.83–17.00) being 4.69 mm (SD 2.09, range 3–17) for smokers versus 4.19 mm (SD 1.08, range 2.83–9.5) in non-smokers (*p* = 0.086). Table 6 gives an overview of the distribution of implants with peri-implantitis between smokers and non-smokers for both jaws.

When taken jaw into account, implants placed in patients with smoking habits experienced a 2.6 higher risk in developing peri-implantitis compared to the implants placed in non-smokers. This difference was borderline non-significant (*p* = 0.053). When comparing jaws, 22.2% and 11.6% of the implants placed in the mandible experienced peri-implantitis in smokers and non-smokers, respectively. Regarding the upper jaw, this was 34.4% versus 9.8%, respectively. These differences were found to be statistically non-significant (*p* = 0.228 and *p* = 0.127). Similarly, no statistically significant difference was present between maxilla and mandible in each group (*p* = 0.481 for smokers and *p* = 0.757 for non-smokers).

## 4. Discussion

This prospective, long-term follow-up study was performed in the setting of private practice. As such, all patients in need of dental implants, irrespective of their medical condition, habits, or socio-economic background, were included. This patient’s inclusion approach may differ from many other clinical studies, whereby often so-called convenience patient samples or rather limited number of cases are treated in an academic teaching facility [50]. The practice-based case selection may imply a more truthful representation of the general population. The present study evaluated the long-term 10-years success of dental implants in relation to the patient’s smoking habits. Smoking status was obtained by self-reporting, which might be not completely trustworthy because of the known fact that patients underreport or deny their smoking behavior [51]. A patient was counted as a smoker if it was mentioned in the patient file, irrespective of the type of smoking or the number of cigarettes a day. In other words, there was no distinction between a light or heavy smoker. Furthermore, patients might cease smoking for a while since the day of implant placement or might have diminished fully or temporally during a certain period. However, as mentioned by Vervaeke et al. (2012) [32], smoking at the time of implant placement was seen as the decisive factor because the initial process of bone healing and soft-tissue healing was directly affected.

The literature is scarce, concerning the long-term effect of smoking on minimally 5 years’ survival of implants with moderately rough surfaces. A recent systematic review, investigating the long-term effect of surface roughness and patient factors on peri-implant bone loss, has included 87 papers out of 2566 reporting on mean bone loss and implant survival with at least 5 years of follow-up [52]. Some of these studies, however, discuss mean bone loss and implant survival based on a rather limited number of implants and patients. Patient compliance and motivation to participate for long-term follow-up is often a difficulty, which may lead to a relatively high drop-out number over time. A recent study concerning implant survival of a large number of implants with a moderately rough surface, placed in the edentulous jaw, shows 10 years’ CSRs of 91.9% for the maxilla and 96.1% for the mandible, with a higher risk for implant failure for the maxilla [53]. A similar study, considering partially edentulous jaws, has shown a 10 years’ CSR of 95.2% [54].

The present study reported an overall survival of 92.7% on the implant level and 82.6% on the patient level after 10 years. This was in accordance with other studies, evaluating the same or similar surface-modified implant systems reporting survival rates of 93.4% up to 100% (Table 7) [55,56,57,58]. 

Compared with the initially reported implant survival rate of 98.3% after 24–58 months, implant failure over time was limited but did not present a completely steady-state. Based on the absolute survival rates, smokers were at 2.5 times higher risk to experience implant failure compared to non-smokers. This number was in agreement with the short-term study by Vervaeke and co-workers (2012) and the meta-analysis by Strietzel and coworkers (2007) [32,71]. Smoking has been identified in the literature as a predictor of implant failure [18]. The maxilla seems more prone to implant failure in smokers, with 5 times higher failure frequency in the present study, in contrast to the mandible with practically no difference in failure [8,17]. Both early and late implant failure were reported. The cumulative survival calculation (Figure 2 and Figure 3) showed that, in this study, non-smokers experienced most implant loss before prosthetic loading, while smokers experienced more late failure. The fact that early failure was only seen in non-smoking patients seemed contradictory to the literature, where smoking habits are linked to a higher risk for early implant failure, especially in the maxilla [27,72,73,74,75,76]. A possible explanation is the relatively lower number of smoking patients included in the study (13.2%). On the other hand, the reported higher late implant failure seemed consistent with the literature, identifying smoking as a risk factor in time [18,74,75,77]. Based on the CSR, smokers showed a 2.7 higher implant failure rate, which seemed consistent with a 3.7 to 4.0 times higher failure reported in the literature [17,32].

Bone loss beyond the reference point was calculated on the patient level because of the systemic character of smoking and on the implant level to avoid masking clinical complications in the case of multiple implants within the same individual. An overall mean bone loss was found of 0.97 mm (SD 1.79) on the implant level and 0.90 mm (SD 1.39) on the patient level after an average of 11.38 years of function. Bone level changes of moderately rough implants are reported to be in the order of 0.18–1.8 mm in the long-term [52,55,56,62,63,64,76]. A systematic review has reported a mean bone loss of 1.01 mm [52]. Many papers included in this review did not take initial bone remodeling into account because baseline radiographs were lacking. As such, the bone loss reported in this review is an underestimation of the true total bone loss. In our study, the baseline radiograph was taken immediately after implant placement. Hence, the mean bone loss of 0.97 mm included both initial bone remodeling as well as a functional bone loss after 10 years.

Smoking has been identified as a predictor of (late) peri-implant bone loss [18,55]. The present study showed a significantly higher peri-implant bone loss in smokers compared to non-smokers. In regard to the jaw of treatment, only in patients with smoking habits, the maxilla showed a significantly higher bone loss. This was in accordance with other studies [15,17,18,29,32,55,78]. The maxilla seems more susceptible to the detrimental effect of smoking, with a time-dependent effect on peri-implant bone loss [7]. A more intense contact between the palate and tobacco smoke, less cortical bone, often jeopardized bone quality, and less protection by the tongue may also explain the difference with the mandible [17]. An imported notification is the synergistic effect of smoking with other risk factors, especially the combination of periodontal pathology and smoking. A recent study has shown an estimated extra bone loss of 2.3 mm after 9 years of follow-up in smokers with a history of periodontitis [55]. With 2 mm as a threshold for implant success, smoking habits in combination with a history of periodontal disease have yielded a 45.3% success vs. 100% for non-smokers with no history of periodontitis [55]. Interaction between different variables (such as age, gender, systemic disease, the jaw of treatment, implant features, loading protocol, prosthetic reconstruction, recall compliance) may be possible. In the present study, only one patient had (regulated) diabetes, and one patient started oral bisphosphonates several years after implant treatment. Because this concerned only one patient for each condition, this was not further investigated. The present study did not use a multivariate analysis to correct these factors because of the relatively small study population. Future research should investigate further in a multivariate way on these different factors in large study populations with long-term follow-up.

Whereas smoking is strongly identified as a risk factor in the literature for periodontal pathology, inconclusive evidence exists regarding smoking as a risk factor or risk indicator for peri-implantitis [79,80]. Some studies have observed a strong association [16,81,82,83,84], but the majority have failed to identify smoking as a risk factor for peri-implantitis. The present study showed a significantly higher peri-implant bone loss in smokers, suggesting a higher chance of developing peri-implantitis. In accordance with this statement, the present study observed a 2.6 higher risk for smokers to develop peri-implantitis (based on the proposed criteria by Berglundh et al. regarding bone loss, BOP, and PPD [49]). However, this finding was not statistically significant, probably due to the relatively small number of smoking patients in this study.

## 5. Conclusions

Implants adapted with a fluoride-modified surface provided a high 10 years’ survival with limited bone loss. Smokers were, however, more susceptible to peri-implant bone loss and experienced a higher rate of implant failure, especially in the upper jaw. The overall bone loss over time was significantly higher in smoking patients, which might be suggestive for a higher peri-implantitis risk. Hence, smoking cessation should be advised and maintained after implant placement from the perspective of peri-implant disease prevention.

## Figures and Tables

**Table 1:** Life table showing an overview of failures and the overall cumulative survival rate on the implant level.

Year Interval	Number of Implant Loss	Number of Implants Entering the Interval	Cumulative Proportion Surviving at the End of the Interval
0	8	453	0.98
1	0	445	0.98
2	2	445	0.98
3	1	443	0.98
4	2	442	0.97
5	2	440	0.97
6	6	438	0.95
7	2	432	0.95
8	3	429	0.94
9	1	421	0.94
10	6	397	0.91

**Table 2:** Life table showing an overview of failures and the overall cumulative survival rate on the patient level.

Year Interval	Number of Patients with Implant Loss	Number of Patients Entering the Interval	Cumulative Proportion Surviving at the End of the Interval
0	7	121	0.94
1	0	114	0.94
2	1	114	0.93
3	1	113	0.93
4	1	112	0.92
5	1	111	0.91
6	5	110	0.87
7	1	105	0.86
8	1	103	0.85
9	1	102	0.84
10	2	96	0.81

**Table 3:** Life table showing a summary of failures and the overall cumulative survival rate (CSR) in non-smokers and smokers with respect to the jaw (on the implant level).

Year Interval	Non-Smokers						Smokers					
	Mandible			Maxilla			Mandible			Maxilla		
	Number of Implant Loss	Number Entering Interval	CSR	Number of Implant Loss	Number Entering Interval	CSR	Number of Implant Loss	Number Entering Interval	CSR	Number of Implant Loss	Number Entering Interval	CSR
0	5	146	0.97	3	231	0.99	0	35	1	0	41	1
1	0	141	0.97	0	228	0.99	0	35	1	0	41	1
2	2	141	0.95	0	228	0.99	0	35	1	0	41	1
3	0	139	0.95	0	228	0.99	1	35	0.97	0	41	1
4	0	139	0.95	0	228	0.99	0	34	0.97	2	41	0.95
5	0	139	0.95	1	228	0.98	0	34	0.97	1	39	0.93
6	1	139	0.95	3	227	0.97	0	34	0.97	2	38	0.88
7	2	138	0.93	0	224	0.97	0	34	0.97	0	36	0.88
8	0	136	0.93	1	223	0.97	1	34	0.94	1	36	0.85
9	0	136	0.93	1	222	0.96	0	28	0.94	0	35	0.85
10	3	124	0.89	0	210	0.96	1	28	0.88	2	35	0.76

**Table 4:** Implant distribution according to implant diameter and length (implant failure is given between brackets).

Diameter (mm)	Length (mm)						
	8	9	11	13	15	17	Total
**3.5**	19 (3)	4 (0)	18 (2)	37 (0)	35 (1)	0 (0)	113 (6)
**4**	28 (3)	16 (1)	26 (0)	49 (8)	77 (4)	8 (3)	204 (19)
**4.5**	7 (0)	19 (4)	11 (0)	22 (0)	18 (1)	0 (0)	77 (5)
**5**	2 (0)	22 (1)	14 (2)	12 (0)	9 (0)	0 (0)	59 (3)
**Total**	56 (6)	61 (6)	69 (4)	120 (8)	139 (6)	8 (3)	453 (33)

**Table 5:** Overview of the successful implants (with 1 mm and 2 mm marginal bone loss as success criterion) in smokers and non-smokers with respect to jaw location.

	Non-Smokers				Smokers			
	**Bone Loss 10y Post-op ≤ 1 mm**		**Bone Loss 10y Post-op > 1 mm**		**Bone Loss 10y Post-op ≤ 1 mm**		**Bone Loss 10y Post-op > 1 mm**	
**Jaw of treatment**	**Count**	**%**	**Count**	**%**	**Count**	**%**	**Count**	**%**
**Total**	272	81.2	63	18.8	37	59.7	25	40.3
**Maxilla**	165	78.9	44	21.1	14	41.2	20	58.8
**Mandible**	107	84.9	19	15.1	23	82.1	5	17.9
	**Bone loss 10y post-op ≤ 2 mm**		**Bone loss 10y post-op > 2 mm**		**Bone loss 10y post-op ≤ 2 mm**		**Bone loss 10y post-op > 2 mm**	
**Jaw of treatment**	**Count**	**%**	**Count**	**%**	**Count**	**%**	**Count**	**%**
**Total**	297	88.7	38	11.3	43	69.4	19	30.6
**Maxilla**	185	88.5	24	11.5	18	52.9	16	47.1
**Mandible**	112	88.9	14	11.1	25	89.3	3	10.7

**Table 6:** Distribution of implant with peri-implantitis in regard to jaw type and smoking status.

Jaw of Treatment	Non-Smokers		Smokers	
	Count	%	Count	%
**Total**	33	10.5	17	28.8
**Maxilla**	19	9.8	11	34.4
**Mandible**	14	11.6	6	22.2

**Table 7:** Overview of long-term clinical studies concerning mostly moderate rough implant surfaces.

Author	Year	Study Design	Subgroups	Follow-Up (y)	Patients	Implants at Baseline	Implants at Follow-Up	Manufacturer	Surface	Baseline	MBL (mm)	SD BL (mm)	Survival %	Surface Roughness
**Hoeksema et al. [59]**	2016	Prospective	Young	10	105	104	99	Straumann	TPS	Loading	1.2	1.1	97.10	Rough
			Older	10		106	64	Straumann	TPS	Loading	1.2	1.2	93.4	Rough
**Vandeweghe et al. [60]**	2016a	Retrospective	No	7.5	46	211	211	Southern	Mod rough	Placement	1.17	0.49	99.50	Moderately rough
**Vandeweghe et al. [23]**	2016b	Retrospective	Mod rough	14.3		121	121	Southern	Mod rough	Placement	1.73	1.54	97	Moderately rough
			Smooth	14.3	33	76	76	Southern	Machined	Placement	1.41	0.92		Smooth
**Van Velzen et al. [61]**	2015	Prospective	No	10	250	506	367	Straumann	SLA	Placement	1.21	0.94	99.70	Moderately rough
**Cooper et al. [62]**	2014a	Prospective	No	5	19	23	18	Astra Tech	Osseospeed	Placement	0.18	0.79	96.5	Moderately rough
**Vervaeke et al. [55]**	2016	Prospective	No	9	50	320	245	Astra Tech	TiOblast	Placement	1.68	2.08	99.20	Moderately rough
**Cooper et al. [63]**	2014b	prospective	Immediate IT	5	113	55	55	Astra Tech	Osseospeed	Placement	0.43	0.63	95	Moderately rough
			Delayed IT	5		58	58	Astra Tech	Osseospeed	Placement	0.38	0.62	98	Moderately rough
**Donati et al. [64]**	2015	Prospective	No	5	151	161	140	Astra Tech	Osseospeed	Placement	0.32	1.15	95.6	Moderately rough
**Rocci et al. [65]**	2013	Prospective	TiUnite	9	44	66	51	Nobel Biocare	TiUnite	Placement	1.40	/	95.5	Moderately rough
			Machined	9		55	39	Nobel Biocare	Machined	Placement	1.70	/	85.5	Smooth
**Dhima et al. [66]**	2013	Retrospective	No	9	81	81	81	Nobel Biocare	TiUnite	Placement	–0.94	0.99	100	Moderately rough
**Mertens et al. [67]**	2012	Retrospective	No	10.1	14	52	52	Astra Tech	TiOblast	Loading	0.30	0.50	100	Moderately rough
**Renvert et al. [68]**	2011	Retrospective	TiOblast	13	41	80	80	Astra Tech	TiOblast	Loading	0.80	/	/	Moderately rough
			Tiunite	13		84	84	Nobel Biocare	TiUnite	Loading	1	/	/	Moderately rough
**Ravald et al. [69]**	2013	Prospective	TiOblast	5	66	184	170	Astra Tech	TiOblast	Placement	0.70	/	95	Moderately rough
			Machined	5		187	175	Nobel Biocare	Machined	Placement	0.40	/	94.70	Smooth
**Mertens et al. [70]**	2011	Prospective	No	8	17	106	99	Astra Tech	TiOblast	Loading	0.30	0.72	99	Moderately rough
**Windael et al. [56]**	2018	Prospective	No	10	21	105	105	Astra Tech	Osseospeed	Loading	0.49	1.08	100	Moderately rough
